# Pennsylvania

**Published:** 1896-04

**Authors:** 


*Pennsylvania.* The JOURNAL has been very kindly favored with advanced sheets of the report of State Veterinarian Pearson, which is fruitful of valuable suggestions.

After referring to the recent legislation establishing the Live-stock Sanitary Board, he refers to the gigantic figures of one hundred million dollars, representing the live-stock industry of Pennsylvania. Attention is called to the necessity of investigation in the line of ascertaining the causes of certain diseases so disastrous to live stock, and to the science of hygiene, so ill understood, together with the care and management of animals, as of very great importance to our people. Knowledge already gained in the methods of caring for animals, in breeding, rearing, feeding, housing, and utilizing them, should be brought to the attention of those who can profit by them in these days of severe competition and small profits.

The commercial and sanitary aspect of meat- and milk-inspection are referred to, and its influence in determining their lessened consumption is brought forcibly to the minds of the producers, and the value of inspection to all concerned strongly indorsed.

Referring to glanders, and its generally well-known and appreciated danger, he calls to the attention of all the fact that this is true, is due to the widespread information upon the subject, and the need of similar knowledge as regards other equally dangerous diseases of an infectious and contagious character would work equally beneficent results.

Speaking of the losses from osteoporosis and its cause, so ill understood, with many conflicting theories of its cause, as well as its character, that it demands more careful study.

The so-called cerebro-spinal meningitis, and the great losses annually occurring therefrom, with its imperfectly-understood etiology, needs to be more thoroughly investigated under the more advanced methods and facilities of the day.

Until careful investigations are made the true cause of periodic ophthalmia will remain unknown.

The need of very thorough destruction of the carcasses of animals dying from anthrax, or their burial not less than five feet below the surface and away from water-courses or highways, is strongly adverted to. The value of vaccination where the disease prevails extensively, or where infected pasture-fields are to be used, is commended as a safeguard. The thorough disinfection of all articles, places, etc., which might have received blood-stains from diseased animals is strongly advised.

Vaccine in districts where blackleg prevails among cattle is advised.

As to Texas or Southern cattle-fever, which occasionally breaks out through some miscarriage of the very excellent regulations of the Secretary of Agriculture of the United States, should be even less under more watchful care of those interested and charged with the execution of these regulations.

Contagious abortion, another fruitful loss to cattle-owners, is referred to, and the value of thorough disinfection of the genital passages of the cow, the external genitals, and stables strongly indorsed.

Reports of contagious garget in herds are asked for, that the subject may be more thoroughly investigated.

(To be continued.)

State Veterinarian Pearson has provided for the use of the State Veterinary Sanitary Board and its agents the necessary blanks for the proper performance of the work of condemnation, appraisement, and quarantine, when the same become requisite.

In Hatboro Township, Montgomery County, six cows were condemned for tuberculosis, upon the tuberculin-test, suggested by finding a tubercular animal sold to a butcher.

The sum of ten thousand dollars has been set aside by the Auditor-General for the State Veterinary Sanitary Board for use until after the close of the fiscal year, June 1st. For the three months, March, April, and May, herds where tuberculosis may be feared or is known to exist may be examined at the expense of the State upon application of the owner, providing the latter will agree to such precautions and measures as are prescribed by the Board, to control its redevelopment, reintroduction, and perpetuation at such centres or places.

The State Live-stock Sanitary Board have had prepared for the more expeditious performance of their work a series of blanks of great value and importance. On one the cattle-owner, in requesting an examination of his herd, after discovering some evidence of disease, agrees to have the animals disposed of according to the rules and regulations of the Board, and to observe such precautions and measures to prevent reintroduction and redevelopment as it may direct. This blank furnishes the number and kind of animals in herds and how the milk is disposed of, and provides for a tuberculin-test if deemed necessary. Another form, similar in its requirements, provides for a health-certificate being furnished, after examination by an appointed representative of the Board, the owner to pay a reasonable and just charge to such representative. Inspections and tuberculin will be furnished free, if owners will agree to assist in the examinations, separate the tuberculous cattle from the herd until disposed of by this Board, disinfect and correct bad sanitary conditions, discontinue the use of milk and cream from infected cows unless boiled or heated to 185° F. and kept at this temperature for five minutes. A form for record of animals destroyed, with post-mortem lesions recorded, giving sex, age, breed, etc. Another form makes a record of the condemned ones, and provides for payment therefor. A form for report of appraisers; certificates to be furnished owner after inspection of herd, with and without tuberculin-test for tuberculosis, and reporting upon sanitary condition of dairy, stables, yards, etc. A blank for report of examining veterinarians, showing number of animals inspected, sex and age, with description of stable, as to size, ventilation, light, drainage, arrangement of stalls, fastenings of animals, same of yard, method of watering, source of water-supply and character of the same, and location and character of milk-room. Certificate for veterinarian's report of animal or animals reported for examination. A form for report of tuberculin-test, with temperature-chart. A blank form for Secretary of Board's use in delegating directions to local veterinarians to conduct examinations.

